# Gaining First Insights on Secondary Progressive Multiple Sclerosis Patients Treated With Siponimod in Clinical Routine: Protocol of the Noninterventional Study AMASIA

**DOI:** 10.2196/19598

**Published:** 2020-07-24

**Authors:** Tjalf Ziemssen, Olaf Hoffmann, Luisa Klotz, Herbert Schreiber, Martin S Weber, Benedict Rauser

**Affiliations:** 1 Department of Neurology, Center of Clinical Neuroscience Carl Gustav Carus University Clinic University Hospital of Dresden Dresden Germany; 2 Department of Neurology St Josefs-Krankenhaus Potsdam Germany; 3 Department of Neurology with Institute of Translational Neurology University Hospital Muenster Muenster Germany; 4 Neurological Practice Center, NTD & Neuropoint Academy Ulm Germany; 5 Institute of Neuropathology University Medical Center Goettingen Goettingen Germany; 6 Department of Neurology University Medical Center Goettingen Goettingen Germany; 7 Novartis Pharma GmbH Nuremberg Germany

**Keywords:** secondary progressive multiple sclerosis, siponimod (Mayzent), S1P modulator, oral therapy, noninterventional study, clinical routine, real-world evidence, disability, cognition, multiple sclerosis, drug therapy, chronic disease

## Abstract

**Background:**

A high proportion of patients with relapsing remitting multiple sclerosis convert to secondary progressive multiple sclerosis (SPMS) characterized by irreversibly progressing disability and cognitive decline. Siponimod (Mayzent), a selective sphingosine-1-phosphate receptor modulator, was recently approved by the European Medicines Agency for the treatment of adult SPMS patients with active disease, as evidenced by relapses or magnetic resonance imaging features of ongoing inflammatory activity. Approval by the Food and Drug Administration covers a broader range of indications, comprising clinically isolated syndrome, relapsing remitting multiple sclerosis, and active SPMS. However, treatment effects of siponimod have not been assessed in a structured setting in clinical routine so far.

**Objective:**

The objectives of AMASIA (imp**A**ct of **M**ayzent [siponimod] on second**A**ry progressive multiple **S**clerosis patients in a long-term non-**I**nterventional study in Germ**A**ny), a prospective noninterventional study, are to assess the long-term effectiveness and safety of siponimod in clinical routine and to evaluate the impact of disease burden on quality of life and socioeconomic conditions. Here, we report the study design of AMASIA.

**Methods:**

Treatment effects of siponimod will be evaluated in 1500 SPMS patients during a 3-year observational phase. According to the genetic polymorphism of CYP2C9, the initial dose will be titrated to the maintenance dose of 1 mg (CYP2C9*1*3 and *2*3) or 2 mg (all other polymorphisms of CYP2C9 except *3*3, which is contraindicated) taken orally once daily. Primary endpoint is the 6-month confirmed disability progression, as assessed by a functional composite endpoint comprising the Expanded Disability Status Scale and symbol digit modalities test to take appropriate account of cognitive changes and increase sensitivity. Further measures including multiple sclerosis activity data; assessments of functional domains; questionnaires addressing the patients’, physicians’, and relatives’ perspectives of disability progression; cognitive worsening; quality of life; and socioeconomic aspects will be documented using the multiple sclerosis documentation system MSDS3D.

**Results:**

AMASIA is being conducted between February 2020 and February 2025 in up to 250 neurological centers in Germany.

**Conclusions:**

AMASIA will complement the pivotal phase III–derived efficacy and safety profile of siponimod with real-world data and will further evaluate several individual treatment aspects such as quality of life and socioeconomic conditions of patients and caregivers. It might help to establish siponimod as a promising option for the treatment of SPMS patients in clinical routine.

**International Registered Report Identifier (IRRID):**

DERR1-10.2196/19598

## Introduction

Most patients with multiple sclerosis (MS), a disabling chronic inflammatory disease of the central nervous system (CNS), initially experience episodes of focal neurological symptoms interspersed by periods of remission (relapsing remitting multiple sclerosis [RRMS]) [1]. Over time, 60% to 90% of RRMS patients convert to secondary progressive multiple sclerosis (SPMS) that is characterized both by an irreversibly increasing disability, with or without new magnetic resonance imaging (MRI) lesions or relapses [1-3], and by the worsening of cognitive function [4]. Cognitive deficits occur more frequently and with higher severity in SPMS patients than in RRMS patients [4].

In individual patients, the risk and timing of the transition from RRMS to SPMS remain largely unpredictable as there are currently no suitable immunologic, clinical, and imaging predictive markers [1,5]. Due to a lack of distinct diagnostic criteria, SPMS is usually diagnosed in retrospect, particularly because pathological parenchymal processes precede clinical symptoms [6,7]. Delineating the clinical profile of RRMS patients at risk of developing SPMS and identifying MRI parameters indicative of SPMS conversion are therefore of primary interest. For this purpose, the real-world evidence study PANGAEA 2.0 [8] has recently been expanded by the additional EVOLUTION study arms [9]. The long-term objective of PANGAEA2.0 EVOLUTION is to establish more accurate and unified diagnostic criteria for the detection of SPMS [10].

As with diagnosis, treatment options for SPMS are limited. Disease-modifying therapies indicated for RRMS as well as investigational RRMS treatments have failed to prevent or slow down disability progression in progressive MS in general [11] and, in particular, in SPMS [5,12-15]. The European Medicines Agency (EMA) has approved only interferon β-1b specifically for SPMS with superimposed relapses [13]. Until now, no immunomodulatory and immunosuppressive drugs have been available for the treatment of SPMS patients without superimposed relapses [16,17]. Siponimod (Mayzent) was approved by the EMA in 2020 for the treatment of adult SPMS patients with active disease, as evidenced by relapses or MRI features of inflammatory activity, commonly interpreted as gadolinium (Gd)-enhancing T1 lesions or active, new, or enlarging T2 lesions [18]. In 2019, the Food and Drug Administration had already approved siponimod with a broader label for the treatment of relapsing forms of MS, including clinically isolated syndrome, relapsing-remitting disease, and active secondary progressive disease, in adults [19].

Siponimod has been developed to selectively modulate the two sphingosine-1-phosphate (S1P) receptor (S1PR) subtypes S1PR1 and S1PR5, which are expressed on lymphocytes (S1PR1), astrocytes, oligodendrocytes, and neurons (S1PR1 and S1PR5) [20]. S1P interaction with S1PR1 expressed on autoreactive lymphocytes appears to be a major driver in early MS pathogenesis, because this interaction is essential for the egression of these cells from secondary lymphoid organs and their infiltration into the CNS [21]. Compared to RRMS, peripheral inflammatory infiltration plays a smaller role in SPMS, as reflected by a low frequency of new Gd-enhancing lesions. Instead, neurodegeneration driven by CNS-intrinsic inflammation is regarded as the predominant feature leading to progressive brain volume loss. Preclinical studies have identified direct neuroprotective properties of siponimod mediated through S1PR1 and S1PR5 receptors (ie, the prevention of synaptic degeneration and promotion of CNS remyelination) [22-24]. In this respect, siponimod represents an improvement over fingolimod (Gilenya), an oral S1P analogue targeting all 5 S1P receptors except S1PR2, for use in patients with highly active or rapidly evolving, severe RRMS [25,26]. The higher S1PR-selectivity of siponimod lowers the risk of adverse events (AEs) [27,28], and the shorter elimination half-life [27] allows patients more flexibility (eg, in case of planned pregnancy).

The phase III study EXPAND (EXploring the efficacy and safety of siponimod in PAtients with secoNDary progressive multiple sclerosis; CBAF312A2304) on the efﬁcacy and safety of siponimod in SPMS patients [29] showed three favorable effects of particular importance for SPMS. First, siponimod attenuated inﬂammatory activity in SPMS patients. When compared to placebo-treated patients, more siponimod patients were found free from both Gd-enhancing lesions and new or enlarging T2 lesions. Second, siponimod reduced the risk of disability progression and lowered rates of brain volume decrease indicating that siponimod slows down neurodegeneration. Third, analyses of participants of the EXPAND study revealed significant and clinically meaningful effects of siponimod on cognitive processing speed, as demonstrated by increased scores in the symbol digit modalities test (SDMT) [30]. Compared to placebo, siponimod increased the proportion of patients with sustained SDMT improvement, while the proportion with sustained SDMT deterioration was reduced [31].

**Figure 1:**
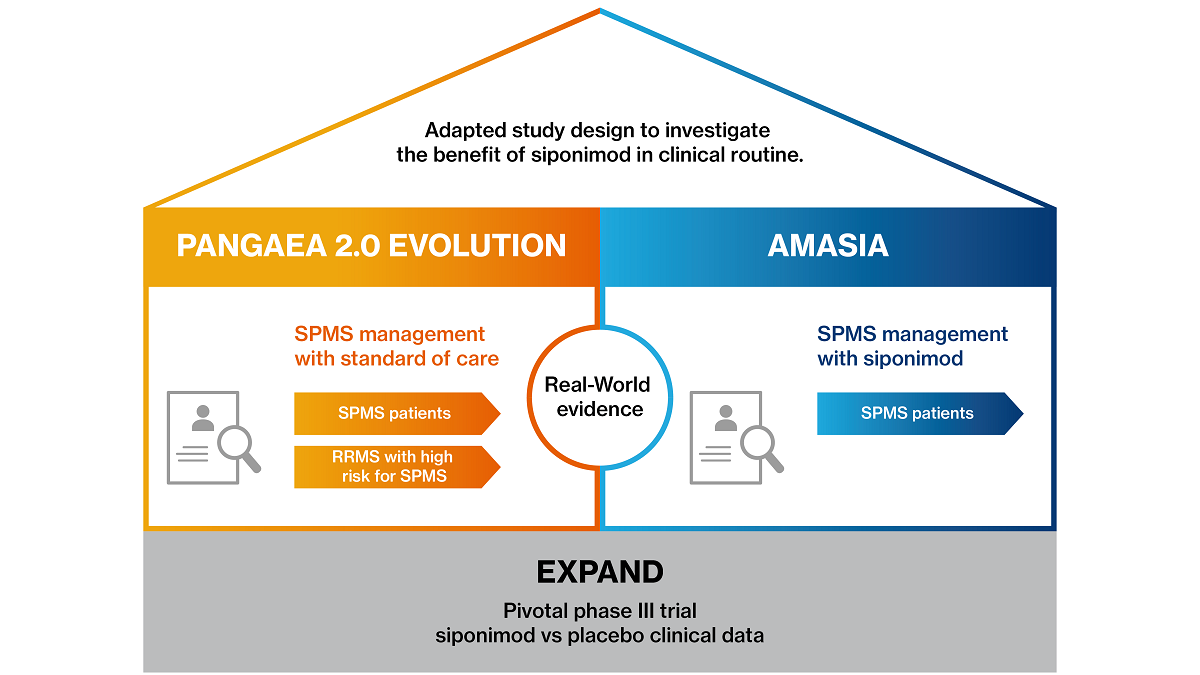
AMASIA within the study framework of siponimod studies. AMASIA: imp**A**ct of **M**ayzent [siponimod] on second**A**ry progressive multiple **S**clerosis patients in a long-term non-**I**nterventional study in Germ**A**ny; EXPAND: EXploring the efficacy and safety of siponimod in PAtients with secoNDary progressive multiple sclerosis [29]; RRMS: relapsing remitting multiple sclerosis; SPMS: secondary progressive multiple sclerosis.

By nature, a pivotal study such as EXPAND with limited follow-up and a narrowly defined patient population cannot address the real-world situation of SPMS patients treated by siponimod in clinical routine [32]. We therefore planned AMASIA (imp**A**ct of **M**ayzent [siponimod] on second**A**ry progressive multiple **S**clerosis patients in a long-term non-**I**nterventional study in Germ**A**ny), a noninterventional study (NIS) to assess the treatment effects of siponimod on SPMS patients in clinical routine. While EXPAND included a representative SPMS population with advanced progression, AMASIA focuses on the much narrower EMA label of SPMS with active disease. In addition to long-term clinical effectiveness and safety of siponimod in real-world situations, AMASIA will assess the effects of siponimod on disease progression of SPMS patients by a wide range of clinical and functional tests and questionnaires concerning the patients’, physicians’, and relatives’ perspectives on disability progression and its consequences, including quality of life and socioeconomic aspects [33]. Furthermore, the study design of AMASIA has been developed following the methodology of PANGAEA 2.0 EVOLUTION [9] to enable a propensity score–matched comparison of SPMS patients receiving either siponimod or standard of care. Therefore, on the one hand, AMASIA will assess real-world treatment effects beyond the clinical data obtained by the phase III study EXPAND. On the other hand, it will allow a comparison of real-world treatment effects of siponimod and current standard of care in SPMS patients (Figure 1). With clinical data of the pivotal trial EXPAND as the base, AMASIA aims to analyze the benefit of siponimod treatment for SPMS patients in clinical routine. Results will be set in relation to the matched study PANGAEA 2.0 EVOLUTION, which analyzes patients with SPMS and high risk for SPMS treated by standard of care [8].

In this paper, we report the study protocol of AMASIA, a multicentric, open-label, prospective NIS. The study started in February 2020 and is planned to continue until February 2025. During the 3-year observational phase, data on 1500 SPMS patients treated by siponimod in up to 250 neurological centers in Germany will be documented.

## Methods

### Study Design

AMASIA is a multicentric, open-label, prospective NIS on adult SPMS patients with active disease as evidenced by relapses or MRI features of inflammatory activity. Clinical diagnosis and decision for siponimod therapy will exclusively be made by the treating physician prior to enrollment and according to clinical routine practice and drug label information [34] to ensure real-world conditions.

Both disability progression, as assessed by an increase in Expanded Disability Status Scale (EDSS) scores, and decline of cognitive processing speed are important indicators of disease burden in MS [35-37]. Therefore, in AMASIA, a novel functional composite primary endpoint combining the 6-month confirmed disease progression (6M-CDP) of the EDSS score and SDMT score is utilized. This composite endpoint has been validated in Germany [38] and will be analyzed after 36 months of siponimod therapy to assess long-term effects of siponimod under real-world conditions. This combined 6M-CDP has been successfully applied for the evaluation of EXPAND patients to increase the sensitivity for SPMS progression of both single measures [38,39]. Confirmed disability progression on EDSS is defined as ≥1.0-point worsening in patients with baseline scores ≤5.0 or 0.5-point worsening in patients with baseline scores >5.0. Concomitantly, a ≥4.0-point SDMT worsening from baseline is considered as clinically relevant [40].

To further evaluate the long-term benefits from siponimod treatment in clinical routine, changes in EDSS (after 36 months) and quality of life scores as well as data on safety and long-term adherence (treatment discontinuation and interruption) will be assessed as secondary endpoints. Adherence will be evaluated on the basis of duration of siponimod intake as well as frequency of discontinuation and dose retitration per patient-year. Additional secondary endpoints are exploratory in nature and concern socioeconomic aspects [41], the characteristics of patients receiving their first dose of siponimod, the timing of therapy initiation, and its impact on disease progression. Furthermore, the utility of progression questionnaires (aligned with the online tool MSProDiscuss) and mobile/digital apps for MS monitoring will be documented [42].

**Figure 2:**
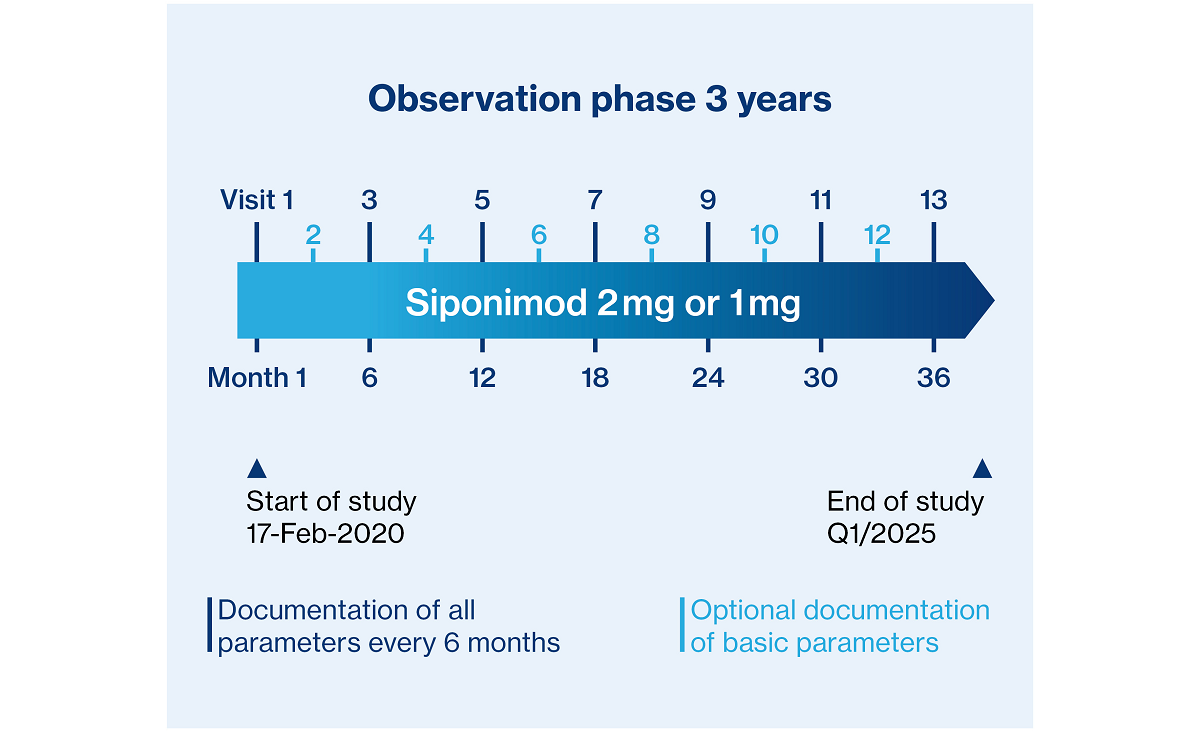
Study design of AMASIA (imp**A**ct of **M**ayzent [siponimod] on second**A**ry progressive multiple **S**clerosis patients in a long-term non-**I**nterventional study in Germ**A**ny), a non-interventional study involving an initial dose titration (visit 1), followed by a 3-year observational phase (visits 2-13).

Because data are obtained in clinical routine, there is no protocol-defined visit schedule during the 3-year observational phase. However, the most probable frequency of visits in clinical routine is every 6 months after the first visit for dose titration. Basically, functional tests will be performed, and questionnaires will be collected in this interval. These regular visits will be complemented by additional visits to obtain basic parameters on clinical characteristics and MS disease activity every 3 months (Figure 2).

AMASIA will be conducted in accordance with the FSA code (voluntary self-regulation for the pharmaceutical industry [43]); joint recommendations of the German Federal Institute for Drugs and Medical Devices and Paul-Ehrlich-Institute on planning, conducting, and evaluating observational studies [44]; and German Association of Research-Based Pharmaceutical Companies recommendations on improving the quality and transparency of NIS [45]. The guidelines for good pharmacoepidemiology practices [46] as well as the STROBE guidelines for the reporting of observational studies will be applied [47]. Study procedures will be carried out in accordance with the Declaration of Helsinki. The Ethics Committee of TU Dresden (Technische Universität Dresden) approved AMASIA.

### Study Population

#### Sample Size

We intend to include 1500 SPMS patients in our study. In the EXPAND study [29], 62.2% of siponimod-treated patients had no disease progression, as determined by the combined 6M-CDP assessed using EDSS and SDMT scores (placebo: 52.3%). Since we expect comparable percentages for AMASIA, a total of 1000 patients completing the 36-month observational phase of AMASIA will be sufficient to estimate the combined 6M-CDP with a precision of 3.0%. With an assumed annual dropout rate of 13%, recruitment of 1500 patients will be required to achieve the intended sample size. Likewise, the same sample size is sufficient to assess EDSS changes over 36 months (secondary endpoint). The calculation is based on the assumed annual dropout rate of 13% and assumed standard deviation of 0.6 for EDSS changes from baseline to month 12 and month 24, as observed in EXPAND [29].

#### Eligibility of Patients

Treatment initiation with siponimod as routine therapy is at the discretion of the treating physician. The decision for siponimod treatment according to drug label information needs to be made before enrollment and independently of study participation. Participants of all genders are eligible if they are 18 years or older, are diagnosed with SPMS, and provide written informed consent. Patients will be excluded due to off-label therapy, pregnancy and breastfeeding, treatment with siponimod before enrollment, and participation in interventional trials or PANGAEA 2.0 EVOLUTION. Patients who do not attend study visits for more than 12 months will exit the study.

### Procedures

#### Dose Titration

After treatment decision and for dose selection, genetic variants of CYP2C9, a cytochrome P450 isoform involved in the metabolism of exogenous compounds by the liver [48], need to be determined in SPMS patients before the first dose of siponimod [34]. These CYP2C9 variants exhibit reduced enzymatic activity, leading to substantially elevated siponimod plasma levels [49,50]. Patients with CYP2C9 genotypes *1*1, *1*2, and *2*2 as well as patients with CYP2C9 genotypes *1*3 or *2*3 undergo a 5-day titration phase. In this titration phase, dosage is gradually increased from days 1 and 2 (0.25 mg) to day 3 (0.5 mg), day 4 (0.75 mg), and day 5 (1.25 mg). The maintenance dose is then 2 mg (CYP2C9*1*1, *1*2, *2*2) or 1 mg (CYP2C9*1*3, *2*3) taken orally once daily. If one dose is missed during the first 6 days of treatment or treatment at maintenance dose is interrupted for 4 or more consecutive days, titration needs to be reinitiated. Siponimod is contraindicated in patients with CYP2C9*3*3 genotype.

In patients with pre-existing cardiac conditions (ie, sinus bradycardia, first-degree or second-degree AV block, or a history of myocardial infarction or heart failure), a first-dose 6-hour monitoring of pulse and blood pressure is recommended, with an electrocardiogram at the beginning and end. Additional monitoring in case of clinically relevant cardiac symptoms as well as appropriate clinical management will be initiated according to the product information [34]. In AMASIA, SPMS diagnosis according to ICD-10, demographic and clinical data including MS-disease and non-MS-disease history, and varicella zoster virus immune status will be obtained during the initial dose titration phase. Varicella zoster virus vaccination of antibody-negative patients is recommended prior to siponimod treatment.

#### Observational Phase

**Figure 3:**
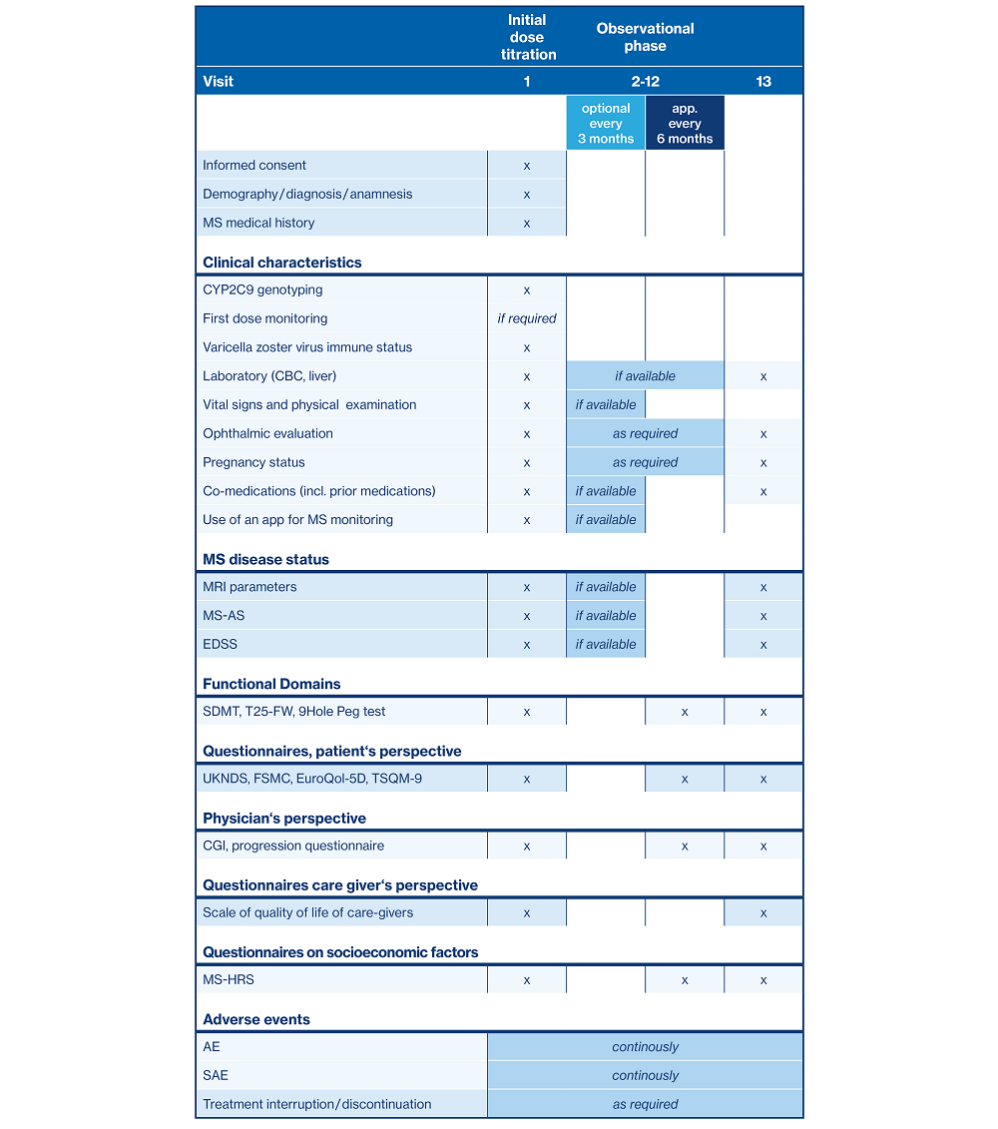
Study visits during AMASIA (imp**A**ct of **M**ayzent [siponimod] on second**A**ry progressive multiple **S**clerosis patients in a long-term non-**I**nterventional study in Germ**A**ny). 9Hole Peg test: nine-hole peg test; AE: adverse event; CBC: complete blood count; CGI: clinical global impression; EDSS: Expanded Disability Status Scale; FSMC: Fatigue Scale For Motor And Cognitive Functions; MRI: magnetic resonance imaging; MS: multiple sclerosis; MS-AS: MS activity scale; MS-HRS: Multiple Sclerosis Health Resource Utilization Survey; SAE: serious adverse event; SDMT: symbol digit modalities test; T25-FW: timed 25-foot walk; TSQM-9: Treatment Satisfaction Questionnaire for Medication; UKNDS: United Kingdom Neurological Disability Scale.

Assessments during the 3-year observational period are depicted in Figure 3. Data on clinical examinations and MS disease status are obtained quarterly, if available. Data on MS disease status include MRI findings concerning number and volume of T2 hyperintense lesions, Gd-enhancing lesions, T1 hypointense lesions, and brain volume changes as well as EDSS and MS activity scale scores. The latter documents time, duration, intensity, treatment, and recovery of relapses.

Results of functional tests and questionnaires are obtained every 6 months (except for the questionnaire on the quality of life of caregivers [51]). Functional tests comprise the SDMT [36], timed 25-foot walk [52,53], and the nine-hole peg test [54] to assess neurological dysfunction and disability of lower and upper extremities. Furthermore, different questionnaires address the patients’ [55-58], physicians’ [59,60], and relatives’ perspectives as well as socioeconomic aspects [61]. Disease progression will be additionally judged by means of a progression questionnaire aligned with the online tool MSProDiscuss, which is intended to facilitate the discussion between physicians and patients about SPMS [42,60].

Occurrence, duration, causal relationship to therapy, counteractive measures to and outcomes of AEs, and treatment discontinuation and interruption will be documented throughout the study. AEs are defined as any unfavorable change in the patients’ pretreatment condition, regardless of their potential relation to treatment and irrespective of whether medication was taken as intended. Serious AEs comprise lethal or life-threatening events leading to persistent or significant disability, congenital anomaly or birth, hospitalizations, and other medically significant events that compromise the safety of patients.

#### Propensity Score–Matched Comparison With PANGAEA 2.0 EVOLUTION

**Figure 4:**
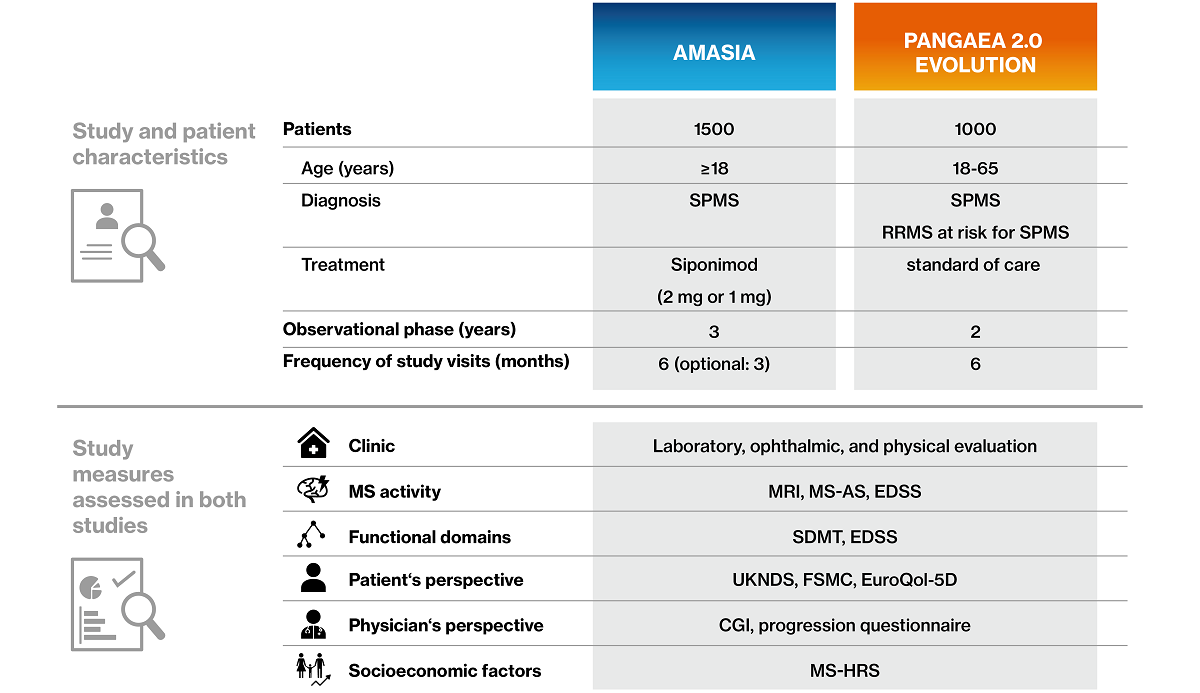
Pairwise comparison of data obtained during AMASIA (imp**A**ct of **M**ayzent [siponimod] on second**A**ry progressive multiple **S**clerosis patients in a long-term non-**I**nterventional study in Germ**A**ny) and PANGAEA 2.0 EVOLUTION, including study design, patient characteristics, and concordant and additional measures of multiple sclerosis (MS) activity, disability progression, functional domains, quality of life, and socioeconomic factors. Standard of care includes current disease-modifying therapy or no treatment at inclusion. CGI: clinical global impression; EDSS: Expanded Disability Status Scale; FSMC: Fatigue Scale For Motor And Cognitive Functions; MRI: magnetic resonance imaging; MS-AS: MS activity scale score; MS-HRS: Multiple Sclerosis Health Resource Utilization Survey; RRMS: relapsing remitting multiple sclerosis; SDMT: Symbol Digit Modalities Test; SPMS: secondary progressive multiple sclerosis; UKNDS: United Kingdom Neurological Disability Scale.

The real-world evidence study PANGAEA 2.0 [8] has been expanded by the EVOLUTION study arms [9] to clinically characterize SPMS patients and RRMS patients at risk for SPMS. In EVOLUTION, patients aged 18 to 65 years and with moderate to severe disability (EDSS 3.0-6.5) are included if they were previously diagnosed with RRMS and have a current diagnosis of SPMS or RRMS at risk for SPMS. Treatment options at inclusion are current disease-modifying therapies or no treatment during the last 12 months. Any treatment option as well as change of treatment are permitted, thereby representing the standard of care for SPMS prior to availability of siponimod. By having aligned the objectives and measures of EVOLUTION and AMASIA, it will be feasible to compare the demographic and clinical parameters as well as the disease impact on functional domains, cognition, quality of life, and socioeconomic parameters. Since patients in EVOLUTION receive standard of care currently available to these patients, the propensity score–matched comparison with data obtained by AMASIA will provide further insights into the long-term effectiveness and safety of siponimod in clinical practice (Figure 4).

### Data Collection and Management Using the Multiple Sclerosis Documentation System MSDS3D

Data will be recorded exclusively online by the physician responsible, using the electronic case report form in the MS documentation system MSDS3D [62,63]. The MSDS3D software combines data documentation with patient management. It now has expanded safety management with regard to the characteristics of different treatments and populations [64,65]. Data will be computerized in a pseudonymous form, and all entries will be automatically controlled for plausibility at the time of data entry and reviewed daily by the database coordinator. Data management processes including data analyses will be conducted by the data management team of the responsible clinical research organization (Winicker Norimed GmbH Medical Research).

### Statistical Analysis

For analysis, descriptive statistics will be primarily used. The full analysis set includes all patients receiving at least one dose of siponimod with at least one available post-dose data recording. Sample statistics such as median, mean (SD), minimum, maximum, 5% percentile, 1st quartile, 3rd quartile, 95% percentile, and number of valid and missing values will be presented in tabular form. Distributions of absolute and relative frequencies will be reported for nominal and ordinal-level data. Incidence rates of all safety outcomes (events per patient-year) will be evaluated for the patient population included. A propensity score matching adjustment, possibly using a caliper-based nearest-neighbor approach as seen in Alsop et al [66], will be used to compare data from AMASIA with data from the PANGAE 2.0 EVOLUTION arm. Possible predictive baseline and prestudy variables to be included in the model are as follows: age at baseline (years), sex, time since diagnosis at baseline (years), treatment at baseline, number of relapses in the past 12 months before baseline, and EDSS score at baseline. A multivariable logistic regression model will be fitted with the treatment arm as the dependent variable. The distribution of propensity scores before and after matching will be analyzed. Using the Medical Dictionary for Regulatory Activities, AEs are categorized according to system organ class and preferred term for individual adverse events (for AEs, serious AEs, adverse drug reactions, serious adverse drug reactions). For all analyses, SAS version 9.2 will be used.

## Results

The study will last from February 2020 through February 2025, with an observational phase of 3 years. Recruitment will end after 2 years or after enrollment of 1500 patients, whichever occurs first (Figure 2).

## Discussion

Here, we report the study design of AMASIA, a NIS prospectively including 1500 SPMS patients to be treated with siponimod in the clinical routine of up to 250 German neurological centers. This study will provide first insights on the benefit of siponimod treatment in clinical routine. During the 3-year observational phase, the long-term effectiveness and safety of siponimod will be assessed under real-world conditions, along with data on the quality of life of both patients and caregivers as well as socioeconomic aspects. Overall, the study aims at obtaining a detailed real-world safety and benefit profile of siponimod, complementing both the phase-III study data of EXPAND [29] and the NIS data of PANGAEA 2.0 EVOLUTION [9] (Figure 1).

The primary endpoint of the study is the 6-month confirmed progression of both disability worsening and cognitive decline, as assessed by a novel composite endpoint comprising 6M-CDP in EDSS and SDMT [38]. In clinical trials, EDSS is the most common outcome measure to determine disability progression in MS and a widely used inclusion criterion to define study populations [67]. However, EDSS alone suffers from limited sensitivity, as it is restricted in assessing impairments of upper extremities and cognition, especially at higher scores [68,69]. Since cognition is more severely and more frequently affected in SPMS patients than in RRMS and even primary progressive MS patients [70-72], the SDMT, indexing the most frequently affected information processing speed domain, was chosen as an additional primary outcome measure. In the assessment of information processing speed, SDMT has been proven to be superior to other tests such as the Paced Auditory Serial Addition Test. Moreover, it is simple and requires less time and assessor expertise to complete [73], and several trials have demonstrated its sensitivity in the evaluation of treatment effects [30,74]. Since Kappos et al [38] showed that 6M-CDP, as assessed by either EDSS or SDMT, is of limited sensitivity when compared to the combined 6M-CDP considering both, we decided to employ this novel composite endpoint to increase sensitivity for changes and therapeutic effects of siponimod.

Additional measures aim at completing the picture of siponimod-treated SPMS patients in clinical routine (Figure 3). The clinical disease course will be characterized by MRI, EDSS, and MS activity scale scores. Since EDSS alone does not comprehensively reflect the disability status of patients, long-term effects on disability of lower and upper extremities are determined by the timed 25-foot walk and nine-hole-peg test, respectively. Questionnaires will capture data on symptoms and disease progression, perceived from the perspective of patients, physicians, and relatives. Importantly, the pairwise comparison with data obtained by PANGAEA 2.0 EVOLUTION [8,9] will extend the safety and efficacy profile of siponimod in clinical routine. Comparability is ensured by sample size, population characteristics, length of observational period, visit interval, and test selection (Figure 4). The combination of data obtained by both NIS will extend the efficacy and safety profile of siponimod obtained by the pivotal phase III study EXPAND [29]. In AMASIA, patient selection is less restricted in terms of age and not restricted in terms of comorbidities, EDSS status at baseline, and history of relapses before enrollment. Furthermore, while EXPAND included a much broader SPMS population, AMASIA concentrates completely on the siponimod EMA-label population with active disease. Therefore, AMASIA will better represent the real-world population of siponimod patients with supposedly earlier stages of SPMS.

AMASIA might provide additional mechanistic information on both the anti-inflammatory effects of siponimod (ie, fewer Gd-enhancing lesions and new or enlarging T2 lesions) and the prevention of neurodegeneration. Traditionally, neurodegeneration during MS pathogenesis is considered to be the consequence of inflammatory attacks mediated by the infiltration of leukocytes in the CNS and the release of inflammatory mediators. However, recent publications suggest that inflammation and neurodegeneration might be two separate aspects in progressive MS, with diffuse white matter injury and cortical demyelination on a background of meningeal, perivascular, and parenchymal inflammation [75]. Indeed, preclinical studies indicated that siponimod prevents and attenuates neurodegeneration [22,76] and promotes remyelination of the CNS [23]. While the EXPAND study indicated that patients with ongoing inﬂammatory activity (ie, superimposed relapses in the 2 years before enrollment) benefited most from siponimod treatment [29], a recent subgroup analysis suggested that treatment effects on disability are largely independent from that on relapses because siponimod reduced the risk for confirmed disability progression in both relapsing and nonrelapsing SPMS patients [77]. AMASIA might confirm these findings with long-term data from clinical routine.

As AMASIA is an observational NIS, it might be associated with some limitations. The lack of randomization to siponimod treatment and the heterogeneity of patients in real-life settings might limit robust conclusions regarding efficacy. However, propensity score–matched comparisons of SPMS patients receiving either siponimod (AMASIA) or standard care (PANGAEA 2.0 EVOLUTION) are thought to assess real-world treatment effects and, therefore, complement phase III data on efficacy. Another limitation to be considered is the potential incompleteness of data obtained during visits not following a predefined visit schedule. Obviously, this risk must be assumed to exist, but the intended systematic, standardized data collection, along with regular monitor visits to ensure data integrity, might be sufficient to minimize bias resulting from incomplete data.

In summary, AMASIA will be the first longitudinal study expanding the approval efficacy and safety profile of siponimod with real-world data. Data collected from this study are differentiated from the phase-III study on one hand and from pure registry studies on the other, through the use of the specialized MS management software tool MSDS3D. In particular, the comparison with data obtained from patients of the PANGAEA 2.0 comparator arm EVOLUTION, who are treated according to the current standard of care, will provide even deeper insights into the long-term effectiveness and safety of siponimod as well as its effects on the quality of life and socioeconomic conditions of SPMS patients. After the United States [19], Germany is the second country in which patients have access to commercially available siponimod. Therefore, the results of AMASIA will be particularly helpful for physicians in other countries to expand the long-term efficiency and safety profile of siponimod and to establish a promising treatment option for SPMS patients.

## Abbreviations

6M-CDP: 6-month confirmed disease progression
9Hole Peg test: nine-hole peg test
AE: adverse events
AMASIA: impAct of Mayzent [siponimod] on secondAry progressive multiple Sclerosis patients in a long-term non-Interventional study in GermAny
CBC: complete blood count
CGI: clinical global impression
CNS: central nervous system
EDSS: Expanded Disability Status Scale
EMA: European Medicines Agency
EXPAND: EXploring the efficacy and safety of siponimod in PAtients with secoNDary progressive multiple sclerosis
FSMC: Fatigue Scale for Motor and Cognitive Functions
Gd: gadolinium
MRI: magnetic resonance imaging
MS: multiple sclerosis
MS-AS: MS activity scale score
MS-HRS: Multiple Sclerosis Health Resource Utilization Survey
NIS: noninterventional study
RRMS: relapsing remitting multiple sclerosis
S1P: sphingosine-1-phosphate
S1PR: S1P receptor
SAE: serious adverse event
SDMT: symbol digit modalities test
SPMS: secondary progressive multiple sclerosis
T25-FW: timed 25-foot walk
TSQM-9: Treatment Satisfaction Questionnaire for Medication
UKNDS: United Kingdom Neurological Disability Scale
